# COVID-19 Pandemic and Ophthalmologists

**DOI:** 10.4274/tjo.galenos.2020.50215

**Published:** 2020-06-27

**Authors:** Joob Beuy, Viroj Wiwanitkit

**Affiliations:** 1 Sanitation 1 Medical Academic Center, Bangkok, Thailand; 2 Dr DY Patil University, Pune, India

Dear Editor,

We would like to share our ideas on “The COVID-19 Pandemic: Clinical Information for Ophthalmologists”.^1^ Bozkurt et al.^1^ discussed several issues related to COVID-19 and ophthalmologists. The general ophthalmologic practice during the COVID-19 pandemic is similar worldwide. In our country, Thailand, the second country to which the disease spread, the postponement of unnecessary ophthalmic procedures is set.^2^ We agree that ophthalmologists are at risk to get COVID-19 infection from occupational work. Nevertheless, there is no report on COVID-19 among ophthalmologists. This might imply that we have good prevention or the ophthalmologist medical service has less risk than other types of medical care service. The use of full protective equipment is necessary and the universal precautions must be used for all patients.^3^ Finally, there is limited mention that patients can also get COVID-19 from medical personnel. The holistic prevention system should be applied in ophthalmology wards.
